# Ventricular assist device selection: which one and when?

**DOI:** 10.3325/cmj.2014.55.596

**Published:** 2014-12

**Authors:** John M. Stulak, Ju Yong Lim, Simon Maltais

**Affiliations:** 1 Division of Cardiovascular Surgery, Mayo Clinic College of Medicine, Rochester, MN, USA; 2 Vanderbilt Heart, Nashville, TN, USA

## Abstract

Advances in mechanical circulatory support have significantly expanded the treatment options for patients with heart failure, whether acute or chronic. There are numerous devices available that offer patients short-, intermediate-, and long-term duration of support depending on their clinical needs and cardiac recovery. Each device has its own technical considerations and the decision which device to use depends on several factors, including what is available, the degree of support required, and expected duration of support. Additional issues that need to be considered in choosing level of support include right heart function, respiratory failure, and multi-organ derangements. A widespread availability of short-term ventricular assist devices and timely institution for effective hemodynamic support will translate into improved patient outcomes whether that is successful transfer to a tertiary care facility or recovery of inherent cardiac function. Implantable ventricular assist devices have and will continue to evolve into smaller and more durable devices, and the future for patients with advanced heart failure looks ever-more promising.

The limited efficacy of medical therapy to treat end-stage heart failure (ESHF), shortage of available donor hearts, and the increasing number of patients who do not qualify for transplant candidacy despite worsening heart failure have all fueled the evolution of mechanical circulatory support. Currently, long-term implantable left ventricular assist devices (LVAD) have significantly expanded treatment options for patients with ESHF as a bridge-to-decision, recovery, transplant, or destination therapy (DT) (1). Mirroring the progress and success of long-term LVADs, advances have similarly been made in the use of short-term VADs for the treatment of cardiogenic shock, whether in the cardiac catheterization laboratory or the post-cardiotomy setting (2). However, despite improving technology, there is still a growing need for wider application of these short-term devices, especially in non-tertiary care centers where lack of access to this technology results in delayed referral and transport to advanced centers and potentially compromises patient outcome. In this setting, there is still a question as to the ideal support device, duration of support, and timing to bridge these critical patients to a longer-term device. Device selection in these circumstances depends on several factors, including the degree of support that is required acutely, logistical/technical considerations, concomitant issues besides left ventricular dysfunction (especially right ventricular or pulmonary dysfunction), and what is available. We review current devices approved by the Food and Drug Administration for short-, intermediate-, and longer-term support, as well as examine the critical issues to be considered with selecting a device. These include timing of support, varying technical specifics of each device, and expected duration of support. In addition, we provide the advantages and disadvantages of each device and provide an algorithm for decision-making and device selection in these patients.

## Cardiogenic shock and short-term devices

The immediate goal for patients in cardiogenic shock is the re-establishment of normal hemodynamics and restoring vital organ perfusion. Cardiogenic shock can occur as a result of myocardial ischemia, valvular heart disease, myocardial disorders (ie, myocarditis), or mechanical complications of myocardial infarction. Ideally, left ventricular support in this setting should be easy to institute, able to rapidly stabilize the patient, able to be transported with the patient, and allow adequate duration of support to address organ derangements and evaluation of neurologic status. A common approach in these patients is conservative, stepwise escalation of support before committing the patient to a more effective mechanical circulatory support (MCS), ie, maximal inotropes, followed by intra-aortic balloon pump, then followed by consideration of VAD therapy. This approach arguably poses a significant delay in reversing malperfusion in these critically ill patients.

Percutaneously implanted devices include the Impella 2.5 and 5.0 (Abiomed Inc., Danvers, MA, USA), TandemHeart (Cardiac Assist, Inc., Pittsburgh, PA, USA), and extracorporeal membrane oxygenation (ECMO). The Impella, a microaxial flow pump, is inserted through a 13-French catheter in the femoral artery and placed across the aortic valve. The Impella 2.5 has been successfully used for left ventricular support during high-risk coronary intervention and for patients with myocardial infarction and cardiogenic shock (1). The Impella 5.0 can be placed percutaneously or, more commonly, through a cutdown for femoral artery access; this device received FDA approval in 2009. Both Impella devices (2.5 and 5.0) provide direct left ventricular unloading with either 2.5 or 5.0 L/min of flow, respectively. Disadvantages of the Impella device include limited availability, relatively short duration of support, frequent cannula dislodgement, lower extremity ischemia, and difficulty during transport to a tertiary care center (1,2).

The TandemHeart is another percutaneously placed VAD. The inflow cannula (17 French) is placed into the femoral vein and advanced into the left atrium under fluoroscopic guidance by a transseptal puncture. The outflow cannula (15 of 17 French) is inserted into the femoral artery. This pump has been successfully used for high-risk coronary intervention, post-cardiotomy heart failure, bridge-to-bridge (BTB), and bridge-to-transplant (BTT) (1-3). A major disadvantage characteristic to this pump includes cannula migration into the right atrium with resultant profound arterial oxygen desaturation. This device is more easily managed during patient transport.

There is considerable experience with ECMO utilized for the treatment of cardiogenic shock. The major addition to the ECMO circuit is an oxygenator and it is the device of choice when patients have both cardiac and respiratory failure. Cannulation depends on the clinical situation; central cannulation is used in the post-cardiotomy setting and percutaneous cannulation through the femoral, subclavian, or neck vessels is more commonly used for cardiogenic shock. ECMO can provide full cardiopulmonary support, but disadvantages of this device strategy include bleeding because of the strict need for intense anticoagulation, limb ischemia when peripheral cannulation is employed, incomplete left ventricular decompression, and difficulty during patient transportation if the circuit is not compact and simplified. This type of support can be used as bridge-to-recovery (BTR), BTB, or BTT.

## Intermediate- to longer-term devices

The Biomedicus Bio-pump (Medtronic, Inc., Eden Prairie, MN, USA) is a centrifugal pump that has been in use since the 1980s. One major advantage of this pump is its widespread availability in most centers that perform cardiac surgery. It is easy to implant and transport patients on this system. However, this pump is prone to fibrin formation in the circuit, and other types of circulatory devices have emerged.

The Abiomed BVS5000 is a pneumatically-driven assist device which consists of a filling chamber (fills passively) and blood returns to the patient via a pumping chamber. Pulsatile flow is not usually in concert with native cardiac contraction and can produce flows up to 6 L/min. The pump head requires exchange every week even with adequate anticoagulation. This device can similarly be used as support until weaning, and as a bridge to longer-term implantable VAD, or BTT.

A newer external VADs based on improved centrifugal technology is the CentriMag (Levitronix, Waltham, MA, USA). Patients with CentriMag can be supported until weaning, as a bridge to a longer-term implantable VAD, or transplant (1). Anticoagulation is required, and if fibrin deposits form in the circuit or pump head, the pump can be exchanged. Each of the devices mentioned above can be used for single or biventricular support. The centrifugal pumps could be combined with a membrane oxygenator to provide effective cardiopulmonary support as an ECMO circuit, albeit limited in duration.

## Long-term implantable devices

As previously discussed, LVAD selection mainly depends on the indication for mechanical circulatory support. In the United States, long-term, implantable ventricular assist devices have been approved for clinical use on two indications, either as BTT or DT. This section focuses on long-term implantable devices, which have received FDA approval for these clinical scenarios.

The clinical indications for use of implantable ventricular assist devices as BTT mainly parallel the eligibility criteria for cardiac transplantation, but its primary therapeutic aim is reducing transplant wait list mortality. Various VADs based on pulsatile pump technology were approved for BTT; these included both paracorporeal pumps such as the Thoratec PVAD (Thoratec Inc., Pleasanton, CA, USA) and the Berlin Heart Excor (Berlin Heart AG, Berlin, Germany), and implantable devices such as the HeartMate XVE and Novacor VAD (WorldHeart Inc., Oakland, CA, USA). More recently, The HeartMate II, an axial flow pump, has proven to be effective in improving functional status, quality of life, and survival in those who clinically deteriorated while awaiting transplantation (4). The third generation centrifugal flow pump technology currently utilized in clinical practice is the HeartWare HVAD, which is FDA-approved for BTT and is under study protocol for DT.

On the other hand, the majority of patients with advanced ESHF simply do not become eligible for heart transplantation due to the limited number of suitable donor hearts. The HeartMate XVE was the first device FDA approved based largely on the results of the REMATCH trial in which transplant-ineligible patients were randomized to either optimal medical management or LVAD therapy (5). At 1 and 2 years, there was a highly significant survival benefit for patients receiving LVAD therapy compared to optimal medical management. Also the quality of life was significantly improved in those supported on LVADs. Despite gaining FDA approval, widespread implantation did not occur because of its large size, specialized patient care requirements, and numerous technical issues. Sepsis and LVAD device failure accounted for the majority of deaths, and high LVAD failure rates remained problematic within 2 years (6). Commonly accepted indications (Centers for Medicare and Medicaid Services criteria) include an ejection fraction <25%, peak oxygen consumption of <14 mL/kg/min, New York Heart Functional Class IIIB or IV, or dependence on either intra-aortic balloon or inotrope therapy. Most patients simultaneously undergo heart transplant evaluation prior to LVAD implantation and based on preoperative testing, they are labeled as either BTT or DT. In some instances, transplant candidacy is unclear and these patients informally receive LVAD therapy as a “bridge to decision.”

LVAD technology has evolved from large, pulsatile pumps into smaller, continuous-flow pumps. The HeartMate 2 has proven to be significantly more durable than the HeartMate XVE, and survival and quality of life were significantly improved in those supported on the new axial flow pumps (7). Currently the HeartMate 2 is the only device that is FDA approved for both BTT and DT, while the HeartWare HVAD is approved for BTT with the DT trial ongoing. Long-term consequences of supporting patients with these continuous flow pumps are yet to be fully understood. Yet, there appears to be an increased incidence of bleeding, perhaps contributed by anticoagulation, acquired von Willebrand deficiency, and/or reduced pulse pressures.

A unique device, which is also FDA approved for BTT, is the pneumatic total artificial heart Cardiowest TAH (SynCardia Systems Inc., Tucson, TX, USA). Indications for TAH implantation differ from those of most VADs. This pump is mainly for patients with severe cardiogenic shock resulting in profound multiple organ derangements, extensive myocardial infarction resulting in biventricular failure, left ventricular failure with extensive intraventricular thrombi, or select cases of primary graft failure or rejection following transplantation.

## Algorithm

As discussed earlier, these implantable devices, which are currently FDA approved for long-term VAD support for either BTT or DT, are typically implanted in patients with chronic heart failure after a thorough evaluation process. However, in patients that are transferred on the aforementioned temporary support instituted for cardiogenic shock or post cardiac surgery, the evaluation process is quite different and their treatment pathway is dependent on several factors (1). Upon arrival, assessment is made of end-organ function, nutritional support is begun, sedation is weaned and thorough echocardiographic evaluation is undertaken. If there is recovery of end-organ function, neurological status and cardiac function, then temporary support can be weaned. If there is end-organ recovery and improvement in neurologic status, but cardiac dysfunction persists, these patients are candidates for HeartMate II implantation. In patients that fail to recover end-organ function with or without cardiac recovery and have poor neurological prognosis, multidisciplinary consideration of temporary VAD support withdrawal is undertaken (1).

Patient presentation and acuity of illness dictate the appropriateness of which device is selected for support. Temporary devices can serve as a bridge in order to stabilize patients and facilitate a work-up for candidacy for long-term implantable devices. This field continues to evolve as do the indications for their use
